# Supplementary material for: Safety and Efficacy of Different Therapeutic Interventions for Primary Progressive Aphasia: A Systematic Review
Source: J Clin Med. 2025 Apr 29;14(9):3063. doi: 10.3390/jcm14093063 (PMC12072502; doi:10.3390/jcm14093063)
Supplement: Supplementary file 1 [file jcm-14-03063-s001.zip › jcm-3585255-supplementary.pdf]

Online Resource: details of PPA therapeutic interventions

Systematic Review

Safety and Efficacy of Different Therapeutic Interventions for Primary Progressive Aphasia: A Systematic Review

Abdulrahim Saleh Alrasheed [1,*], Reem Ali Alshamrani [2], Abdullah Ali Al Ameer [3], Reham Mohammed Alkahtani [2], Noor Mohammad AlMohish [4], Mustafa Ahmed AlQarni [5] and Majed Mohammad Alabdali [5]

[1] Department of Neurosurgery, College of Medicine, King Faisal University, AlAhsa 31982, Saudi Arabia
[2] College of Medicine, Taif University, Taif 21944, Saudi Arabia; reemalshamrani-@hotmail.com (R.A.A.); s43900691@students.tu.edu.sa (R.M.A.)
[3] College of Medicine, King Faisal University, AlAhsa 31982, Saudi Arabia; 221415298@student.kfu.edu.sa
[4] Neurology Department, King Fahad Hospital of the University, Imam Abdulrahman Bin Faisal University, Khobar 34445, Saudi Arabia; nmalmohish@iau.edu.sa
[5] Neurology Department, College of Medicine, Imam Abdulrahman Bin Faisal University, Khobar 34445, Saudi Arabia; mqarni@iau.edu.sa (M.A.A.); mmalabdali@iau.edu.sa (M.M.A.)
* Correspondence: 221414880@student.kfu.edu.sa or abdulrhim2003@hotmail.com

Results

Treatment Gain:

Naming and word-finding for trained and untrained words:

Various therapeutic interventions have demonstrated effectiveness in improving naming and word-finding abilities in individuals with PPA. While transcranial direct current stimulation (tDCS) combined with language therapy, speech-language therapy (SLT), and phonological-orthographic treatments consistently enhance naming accuracy and lexical retrieval, interventions like errorless learning therapy, Lexical Retrieval Cascade (LRC), and semantic feature training show varying degrees of generalization and maintenance over time, with some extending benefits up to six months post-treatment [28,29,34,37,41,43,49,54,58,62,68,76].

tDCS combined with language therapy has demonstrated significant improvement in word retrieval, naming accuracy, and lexical retrieval for trained items, with some studies reporting generalization to untrained words [28,37,38,41,44,78,75,82,83]. Similarly, SLT has shown significant enhancement in word retrieval, extending to both trained and untrained words, with improvements persisting up to six months post-treatment [27,41,47,60,75]. Additionally, phonological and orthographic treatments have yielded notable gains in naming accuracy and lexical retrieval, with evidence of generalization to untrained tasks [37,41,42,50,70]. Errorless learning therapy has also been effective in improving word retrieval, particularly for trained words, with improvements maintained up to three months post-intervention [36,48,66]. LRC approach has produced significant gains in word retrieval, primarily for trained items, with follow-ups extending up to six months [50,63,68,76]. Furthermore, tDCS combined with Constraint-Induced Language Therapy (CILT) has been associated with improved word retrieval, although follow-up data is limited [33].

Semantic Feature Training with tDCS has demonstrated significant improvements in naming accuracy, with maintenance of gains observed at two months post-treatment [54,58]. Rapid Retraining of Items (RRIPP) with Cognitive-Oriented Enhancements (COEN) has also led to improved word retrieval and naming accuracy for trained items, with gains persisting for up to six weeks [46,47]. The Visual Semantic Treatment Approach (VISTA) has shown substantial improvement in word retrieval, with evidence of generalization to untrained items and maintenance of benefits up to six months post-treatment [34,39]. Smartphone-based cognitive and picture-naming therapy has further contributed to improved naming accuracy [30]. Lastly, repetitive transcranial magnetic stimulation (rTMS) has been associated with enhancements in word retrieval in certain studies, although results have been variable [35]. These findings highlight the diverse range of effective therapeutic interventions available for enhancing naming and word-finding abilities in individuals with PPA, with variability in generalization and maintenance of treatment effects.

Spontaneous speech or fluency

Various interventions have shown promise in improving spontaneous speech and fluency in individuals with PPA. While smartphone-based cognitive therapies and tDCS combined with speech therapy enhanced phonemic fluency and speech rate, other approaches like rTMS, VISTA therapy, and structured oral reading therapy contributed to gains in grammatical accuracy, mean length of utterance (MLU), and reduced speech errors, though semantic fluency improvements were less consistent [30,33,34,35,39,48,49,51,54,56,57,63,64].

Smartphone-based cognitive and picture naming interventions, such as those in the study by Joubert (2023), showed that spontaneous speech remained fluent and well-articulated, albeit with occasional word-finding difficulties [30]. For more targeted approaches, the use of HD-tDCS combined with CILT demonstrated improvements in phonemic fluency compared to sham treatment, though semantic fluency did not show significant differences [33]. In addition, phonemic fluency also improved significantly with tDCS combined with speech therapy, as seen in the study by Borrego-Ecija (2023), where the active tDCS group showed greater improvement (0.54 ± 0.62) versus the sham group (0.20 ± 0.55). Meanwhile, other treatments involving tDCS and speech therapy yielded various degrees of improvement, including naming accuracy for trained items [54], speech rate and MLU [48], and spontaneous speech fluency [57].

Further treatments such as rTMS [35] and hf-rTMS [51] showed mixed results, with significant improvements noted post-intervention in rTMS [35] and improved outcomes after real stimulation in hf-rTMS [51]. VISTA therapy [34,39] demonstrated consistent improvements in grammatical errors, MLU, speech rate, and intelligibility across several studies. Additionally, studies involving errorless learning therapy [49] and cueing hierarchy with story retelling [56] also reported improvements in naming abilities, with some treatments resulting in improved naming accuracy for both treated and untreated items [56]. Finally, lexical retrieval interventions [64] and structured oral reading therapy [63] contributed to significant improvements in verbal communication and reduced speech errors during specific tasks.

Auditory verbal comprehension

Various interventions have been effective in improving auditory verbal comprehension in individuals with PPA. Treatments such as tDCS combined with language therapy, SLT, phonological and orthographic treatments, and VISTA have demonstrated significant gains, with some studies reporting generalization to untrained stimuli and sustained improvements for up to six months post-treatment [28,29,30,32,34,37-39,41-44,46-49,50,54,58,63,65,68,70,75-77,82,83].

tDCS combined with language therapy has demonstrated significant improvements in auditory comprehension, particularly for trained items, with some studies reporting generalization to untrained stimuli and maintenance of gains over time [28,37,38,41,44,48,68,75,82,83]. Similarly, SLT has been effective in enhancing auditory comprehension, with evidence suggesting improvements extend beyond trained words and persist for up to six months post-intervention [28,43,49,70,77]. Phonological and orthographic treatments have also shown positive outcomes, particularly in improving comprehension accuracy, with some studies reporting generalization to untrained linguistic contexts [37,41,42,50]. Errorless learning therapy has yielded improvements in auditory comprehension, with maintenance of gains observed up to three months post-treatment, though primarily for trained items [36,48,66]. The LRC approach has resulted in significant gains in auditory comprehension, especially for trained words, with follow-up studies indicating sustained improvements up to six months [50,63,68,76]. Additionally, tDCS combined with CILT has been associated with improvements in auditory comprehension, though long-term follow-up data remains limited [33].

Semantic Feature Training with tDCS has demonstrated significant benefits in comprehension accuracy, with gains maintained at two months post-treatment [54,58]. RRIPP with COEN approach has led to improvements in auditory comprehension, particularly for trained stimuli, with effects persisting for up to six weeks [46,47]. VISTA has shown substantial enhancement in auditory comprehension, with some generalization to untrained items and maintenance of benefits up to six months [34,39]. Additionally, smartphone-based cognitive and picture-naming therapy has been effective in improving auditory comprehension [30]. Lastly, rTMS has been investigated for its effects on auditory comprehension, with variable outcomes reported across studies [35]. These findings underscore the efficacy of multiple intervention strategies in enhancing auditory verbal comprehension in individuals with PPA, with varying degrees of generalization and long-term retention of benefits.

Repetition

Various interventions have shown promise in improving repetition abilities in individuals with PPA. tDCS combined with language therapy and ICAT demonstrated significant post-treatment gains [28,41,44,48,61,68,75,80]. While SLT and errorless learning therapy have also been effective, other approaches such as rTMS and VISTA therapy showed limited or no significant improvements in repetition abilities [29,30,34,35,42,43,49,50,52,55,66,73,76,79].

tDCS combined with language therapy has shown significant improvements in sentence repetition and word-picture verification accuracy across multiple studies [28,48,61,68,75,80]. However, some studies noted no significant improvement in control groups, suggesting that treatment effects may be specific to active interventions. Similarly, tDCS combined with cognitive therapy, particularly ICAT, demonstrated a significant improvement in repetition abilities post-treatment ($p < 0.05$) [41,44]. SLT has been investigated as a non-invasive intervention, with findings indicating that word repetition abilities either improved or remained stable over time, while control groups exhibited no significant changes [28,42,43]. Some participants demonstrated notable improvements in repetition abilities, reinforcing the potential benefit of SLT in maintaining or enhancing language function in PPA. Likewise, errorless learning therapy, as explored in studies by Jokel (2014, 2016) and Themistocleous (2021), showed improvements in repetition abilities, with one study reporting high repetition success in early trials involving words and sentences [49,55,66].

Cognitive and picture-naming therapy results were more limited, with one study reporting normal baseline repetition ability (MT-86 Repetition: 33/33), indicating no significant impairment to begin with [30]. Phonological and orthographic therapy has also been evaluated, with results showing varied improvements in pseudoword repetition (mean = 6.8, SD = 3.0 for svPPA) [42,50,52]. However, studies investigating rTMS combined with language therapy did not find significant improvements in sentence repetition abilities [35].VISTA therapy, though effective in other linguistic domains, showed no significant improvements in sentence and word repetition abilities when compared to control groups [34]. Finally, studies examining the lexical retrieval cascade approach yielded mixed results, with some participants demonstrating improved lexical retrieval scores [73,76,79].

Apraxia of speech

Various interventions have shown promise in improving apraxia of speech (AOS) in individuals with PPA, with VISTA therapy, structured oral reading therapy, and lexical facilitation training (LeFT) combined with tDCS demonstrating notable speech accuracy improvements [34,39,45,52,57,63].

Additionally, tDCS, high-definition tDCS (HD-tDCS), and rTMS have contributed to enhanced speech fluency, word retrieval, and reading efficiency, though outcomes vary depending on the therapy approach [35,53,55,56].VISTA therapy has demonstrated significant improvements in speech accuracy, particularly in reducing speech errors on multisyllabic words, with benefits maintained post-treatment [34,39,45]. Structured oral reading therapy has also shown effectiveness in reducing speech errors in individuals with nonfluent variant PPA (nfvPPA) [52,63]. Similarly, the combination of LeFT with tDCS has led to improvements in lexical retrieval [57].

The application of tDCS in combination with speech therapy has yielded positive outcomes, with a significant reduction in sound duration observed in individuals with AOS and nfvPPA [55]. Additionally, rTMS has been associated with varying degrees of improvement in speech-related tasks, including an increase in word count during spontaneous speech (27.89 ± 34.59), as well as enhancements in reading accuracy (20.55 ± 64.60) and reading efficiency (18.33 ± 57.74) in story reading tasks [35]. HD-tDCS combined with story retelling and cueing hierarchy therapy has also been investigated, showing improvements in spontaneous speech and word retrieval during therapy phases [53,56].

Reading abilities

Phonological and orthographic treatments have been effective in improving reading accuracy across different PPA subtypes, particularly in maintaining written naming accuracy for patients with logopenic variant PPA (lvPPA) [41,42,63]. While errorless learning therapy has shown benefits across multiple PPA variants, rTMS has produced mixed results, with some studies reporting improvements in reading efficiency and others indicating declines in performance post-treatment [35,36,62,66,67]. Studies have reported significant improvements in phonological and orthographic task performance, with particular benefits in maintaining written naming accuracy for patients with lvPPA [41,42,63].

Errorless learning therapy has also been examined as an intervention for reading impairments in PPA, particularly for patients with svPPA, nfvPPA, and lvPPA. Research suggests that this approach enhances error-free learning and contributes to improved word reading accuracy, with significant benefits observed across different PPA variants [36,66,67]. rTMS has shown mixed results in enhancing reading abilities. While certain studies have demonstrated significant improvements in reading accuracy and efficiency following treatment [35,62], some cases reported negative effects, with substantial declines in pre- and post-treatment performance. These findings highlight the variability in response to rTMS and suggest that individualized treatment approaches may be necessary to optimize outcomes.

Cognitive Function

Various interventions have demonstrated improvements in cognitive function in individuals with PPA, with tDCS combined with speech therapy yielding enhanced MMSE scores and cognitive performance, while SLT and phonological treatments showed limited effects [28,38,48,68,80]. Smartphone-based cognitive therapy produced the most significant cognitive gains, highlighting its potential for enhancing language and memory functions [30].

tDCS combined with speech/language therapy resulted in improved MMSE scores across different PPA subtypes, with post-treatment scores ranging between 18 and 28. However, some participants exhibited no significant cognitive changes, while improvements in word-picture verification and cognitive abilities were observed [28,38,48,68,80]. SLT alone showed only slight declines in MMSE scores over time, with no substantial improvements in cognitive measures [29,43,50,42]. Similarly, phonological and orthographic treatment did not yield significant improvements in MMSE scores, with some participants experiencing slight declines, particularly in verbal fluency [50, 52,77]. Lexical retrieval cascade therapy yielded mixed results, with some studies reporting improvements in MMSE scores [45], while others showed declines over six months to one year [63,76]. Smartphone-based cognitive therapy demonstrated the most notable cognitive improvements, with participants achieving MMSE scores of 27/30 and significant gains in multiple cognitive tests, including the MoCA and RAVLT [30].

Generalization and Maintenance of Therapeutic Effects

The long-term retention of treatment benefits varied across interventions, with tDCS and phonological therapy demonstrating sustained effects for several months [28,38,41,42,68]. Smartphone-based cognitive therapy and VISTA therapy, maintained language improvements over six-month follow-ups, supporting their role in prolonged therapeutic impact [30,34,39,45].

The maintenance of treatment effects varied across intervention types. tDCS combined with language therapy demonstrated sustained improvements in trained items, with some studies reporting maintenance of gains for up to three months [28,38,48,68,75,80]. SLT showed long-term maintenance of improvements at six-month follow-ups [29,63], while phonological and orthographic treatment effects persisted for up to 15 months in some cases [37,41,42]. Errorless learning therapy led to the

retention of trained items for up to three months, but maintenance beyond that period was inconsistent [36,66,70]. RRIPP combined with COEN therapy demonstrated some maintenance of trained item improvements, though generalization effects were limited [46,78]. VISTA therapy yielded stable improvements in MMSE and other primary measures over long-term follow-ups [34,39,45]. Smartphone-based cognitive therapy facilitated maintenance of language gains at a six-month follow-up [30], while cueing hierarchy and story-retelling therapy preserved treatment effects for trained items over similar periods [56].

Neuroplastic and Functional Reorganization

Therapeutic interventions induced neuroplastic changes, with tDCS promoting cortical activation in perilesional and contralateral regions, while phonological and SLT approaches enhanced structural connectivity in language-related areas [28,42,48,75,77]. These findings suggest functional reorganization as a key mechanism underlying treatment effects. Neuroplastic changes were evident in multiple intervention studies. tDCS combined with language therapy led to increased cortical activation in perilesional and contralateral homologous regions, supporting neuroplastic reorganization [56,48]. Phonological and orthographic treatments also induced changes in left inferior frontal and superior temporal regions, associated with language processing improvements [42,77]. SLT demonstrated structural changes in the left superior longitudinal fasciculus, reinforcing functional connectivity adaptations in response to therapy [29,75].

Structural and Metabolic Changes

Brain imaging studies revealed structural and metabolic changes following interventions. tDCS combined with SLT was associated with increased gray matter density in the left inferior frontal cortex [80,82]. Errorless learning therapy resulted in improved metabolic activity in the left temporal lobe, while lexical retrieval cascade therapy enhanced functional connectivity in temporal-parietal regions [62,76]. Additionally, VISTA therapy showed improved glucose metabolism in key language-related areas, suggesting metabolic support for language gains [45].

Personalized Treatment

Personalized interventions tailored to specific linguistic deficits yielded greater treatment efficacy. SLT approaches customized to phonological, lexical, or syntactic deficits resulted in improved retention and generalization of treatment gains [29,62]. Smartphone-based cognitive therapy provided individualized adaptive feedback, enhancing participant engagement and compliance [30]. Similarly, cueing hierarchy and story-retelling therapy were most effective when adapted to individual linguistic profiles [56].
